# Auditory stimuli degrade visual performance in virtual reality

**DOI:** 10.1038/s41598-020-69135-3

**Published:** 2020-07-23

**Authors:** Sandra Malpica, Ana Serrano, Diego Gutierrez, Belen Masia

**Affiliations:** Universidad de Zaragoza, I3A, 50018 Zaragoza, Spain

**Keywords:** Computer science, Neurophysiology

## Abstract

We report an auditory effect of visual performance degradation in a virtual reality (VR) setting, where the viewing conditions are significantly different from previous studies. With the presentation of temporally congruent but spatially incongruent sound, we can degrade visual performance significantly at detection and recognition levels. We further show that this effect is robust to different types and locations of both auditory and visual stimuli. We also analyze participants behavior with an eye tracker to study the underlying cause of the degradation effect. We find that the performance degradation occurs even in the absence of saccades towards the sound source, during normal gaze behavior. This suggests that this effect is not caused by oculomotor phenomena, but rather by neural interactions or attentional shifts.

## Introduction

The two most used sensory modalities that help humans perceive extrapersonal space are sight and hearing^1^. While sight is the dominant sensory modality when perceiving the outside world, we rely on hearing to retrieve information for regions of space that we cannot see (i.e., rear space or occluded objects)^2^. The human brain processes the visual information to yield a coherent image. As part of this processing, it has evolved to discard or suppress some of this visual information in order to maintain a stable and congruent vision. This suppression happens consistently: during blinks it usually goes unnoticed thanks to a neural inhibitory mechanism in the brain^3^. For saccades (a quick, simultaneous movement of the eyes between two phases of fixation), our vision remains clear since the blurry images produced by high-speed eye movements are suppressed by the brain^4^. In addition to blinks and saccades, other visual suppression effects exist, triggered by different neural mechanisms^5^.

Sensory cortices of different modalities (visual, auditory, etc.) are anatomically separated. However, several studies show that a multimodal interplay exists even between primary sensory cortices^6^. In particular, crossmodal inhibitory interactions have been found in humans for auditory and visual modalities^7^, and for tactile and visual modalities^8,9^. Several brain imaging studies have also shown crossmodal inhibitory or modulatory cortical responses^10–13^ in what were previously considered unimodal processing areas. The areas where neural suppression occurs are also identified, including parts of the sensory cortices. A deep and comprehensive understanding of crossmodal effects can be leveraged for applications beyond vision science, such as visual computing, immersive environments, or the design of novel display hardware.

In this work, we focus on how sound can degrade visual performance in VR. Despite the recent success of this emerging technology, the viewing behavior and mechanisms triggered by this new medium are not yet well understood^14^. Specifically, we investigate whether the presence of an auditory stimulus can degrade the detection and recognition of visual stimuli that appear in a temporally congruent manner with the auditory stimulus, as compared to the performance in the presence of the visual stimuli only (i.e., without an associated, temporally congruent auditory stimulus). Facilitating effects with spatially congruent modalities have been assessed in several studies^1,15,16^. We thus choose to present sounds in a spatially incongruent manner with the visual stimuli. Moreover, previous work has shown that crossmodal interactions taking place in rear space are often different from those in frontal space, since in rear space we have to rely on sounds to obtain information that cannot be retrieved visually^2^. Hidaka and Ide^7^ showed that white noise bursts can degrade visual performance significantly in laboratory conditions; they used a fixed-head experiment setup in which the visual stimuli were tilted Gabor patches displayed on a conventional monitor. We differ from this previous work in several aspects, which aim to increase our knowledge of the phenomenon, generalize the findings, and bring them closer to their potential application scenarios.

First, we extend the analysis from low-level Gabor patches to realistic environments, including a task of higher cognitive load: We broaden the task from binary recognition in previous work, to detection *and* recognition of five possible visual targets. Crossmodal effects are still present in such higher cognitive load conditions. For example, it has been demonstrated that auditory spatialization can facilitate speech recognition^17^. When stimuli of different modalities are presented in a temporally congruent manner, it has also been shown that attention can be selectively diverted from a target to a secondary speaker^18^. Crossmodal effects in VR can even help to create illusions of different sensory modalities^19^. Second, we explore a wider range of different sounds with varying complexity. While previous work used only white noise, we also analyze pure frequencies, pink and brown noise, and two different sounds with semantic content for a total of six different sound types. Beyond their characteristics (i.e., frequency content), these sounds have been chosen due to how they affect perceptual processes; more details on this can be found in the “Methods” section. In addition, we explore the influence of the type and spatial location of sound, as well as its interaction with the shape and spatial location of the visual target. Further, instead of using a regular monitor, we conduct our experiments in an immersive VR setting. The reason for this is three-fold: First, VR offers a greater control over the conditions of the experiment, increasing reproducibility and repeatability; second, it allows for a more natural exploratory behavior of the subject, including walking around the scene, in contrast with previous approaches that required a fixed head position; and third, auditory-triggered visual performance degradation can find key applications in VR, where control of the user’s attention is a fundamental challenge^20^. Moreover, it has been assumed in the literature that the visual performance degradation is caused by neural inhibitory interactions between the auditory and visual sensory pathways. However, it remains unknown if saccades towards the sound source (and hence saccadic suppression) are related to this effect. To explore this, we record gaze data by means of an eye tracker built into the head mounted display (HMD) and analyze gaze behavior during the experiment. The data and stimuli are available for reproducibility and further analyses at https://smalpica.github.io/visDegradation/.

Our main findings include:

- We find that the visual performance degradation effect is robust even for viewing conditions that impose a higher cognitive load, including natural exploratory behavior. This is important since these factors could potentially affect or mask the inhibitory effects reported in the literature.
- We find a consistent and significant degradation of both detection and recognition of the visual targets regardless of both sound location and the location or shape of the visual target.
- Our gaze data reveals that gaze behavior does not change even in conditions where visual performance decreases significantly, suggesting that the effect is not caused by oculomotor phenomena.

## Results

### Experiment zero (baseline): visual detection and recognition in the absence of auditory stimuli

**Figure 1:**
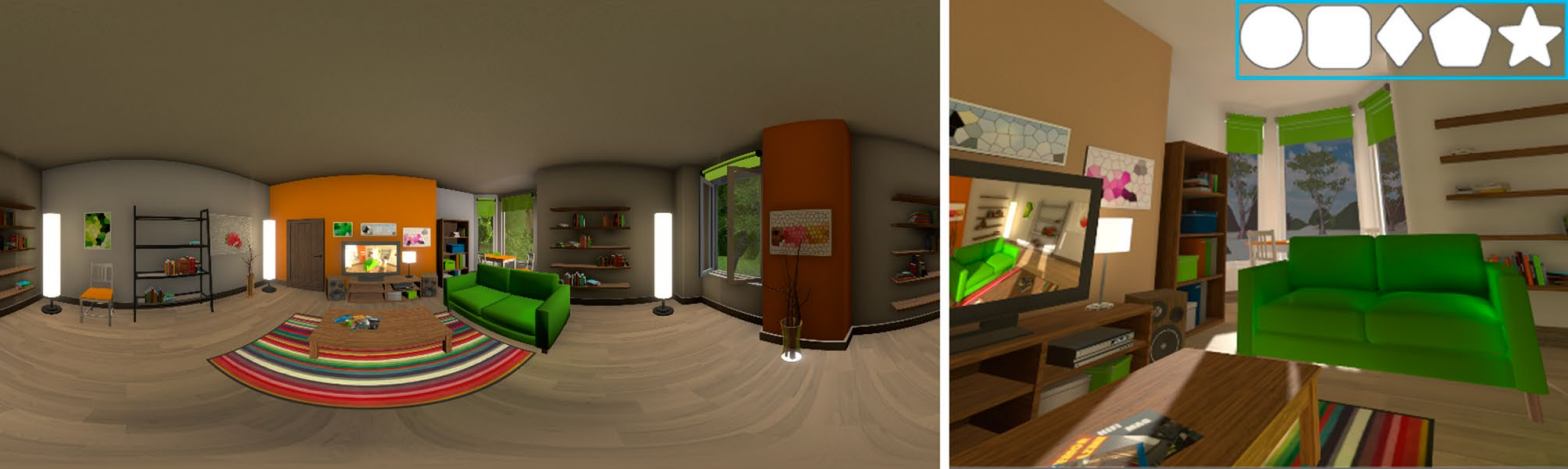
*Left:* 360$^\circ$ panorama of the virtual environment used in the experiments, rendered from the central point of view. *Right:* Representative close-up view of the VE. The inset shows the five different visual targets (a 3.2$\times$ scale is used here for visualization purposes). Users could move freely in a physical space of $4\times 1.5$ m. Scene by Barking Dog for Unity 3D.

**Figure 2:**
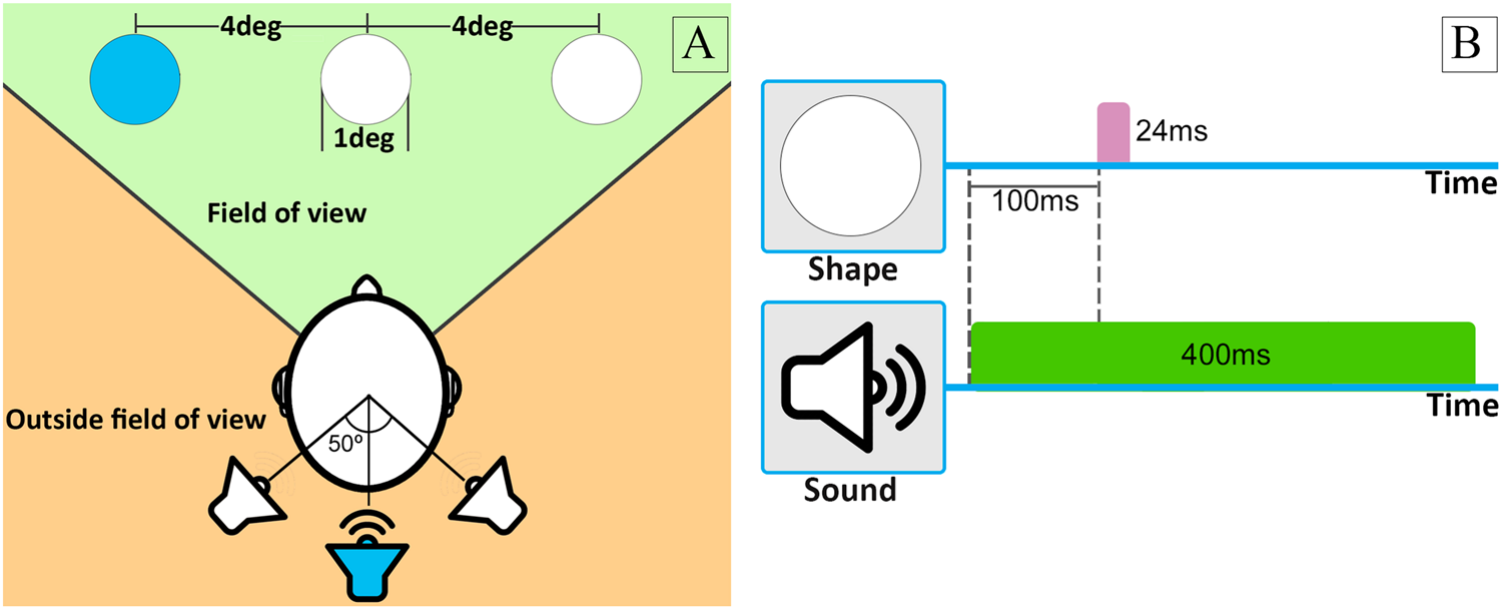
(**A**) Spatial layout of the experiments. The three possible locations of the visual targets inside the participant’s FOV: center, and 4$^{\circ }$ of visual angle to the left and right. The targets subtended a visual angle of 1$^{\circ }$. Auditory stimuli were spatially located outside the FOV, also in one of three possible locations, all of them 0.2 m (Unity distance) from the user position: behind the participant, 50$^{\circ }$ left or 50$^{\circ }$ right. Both the visual targets and the auditory stimuli kept their positions fixed relative to the participant’s head. One possible combination for a *biCond* stimuli is highlighted in blue. (**B**) Temporal layout. Visual targets are shown 100 ms after the sound starts, for a duration of 24 ms. The auditory stimulus lasts 400 ms. Gaze behavior is quantitatively analyzed in those 400 ms to study the relationship between the presence of sound and the visual performance degradation effect.

To ensure that the visual targets were detectable and recognizable we first ran an experiment in the absence of concurrent auditory stimuli. The 360$^{\circ }$ virtual environment (VE) displayed in the VR headset showed a realistic living room as shown in Fig. 1. The visual targets were five different simple shapes (circle, square, rhombus, pentagon, and a five-pointed star), placed at one of three possible locations inside the field of view (FOV) of the subject: center, 4$^{\circ }$ to the left, or 4$^{\circ }$ to the right, always on the FOV equator (see Fig. 2 A, green area). All stimuli were white with a grey outline to help differentiate them from the VE. The subtended visual angle of each visual target was 1$^{\circ }$.

A background auditory context was added, consisting on diegetic, localized audio (sounds from a park coming through the window, and a news podcast playing through one of the speakers near the TV). Each visual target appeared for 24 ms^7^ and the interval between targets was randomly chosen between 5 and 10 s to prevent potential learning effects. The participants had to verbally report each time they saw a visual target, and specify its shape. They were explicitly told to notify the appearance of a target even if they could not recognize its shape. Each participant saw a total of 50 targets. The mean percentage of target **detection** (*binary response; the participant was able to identify the appearance of a target*) was 88.10% (± 4.20%, 2×SEM). Wilcoxon tests were used to check for differences between experiments or between conditions (pairwise comparisons), while GLMM models were used to analyze the influence of the studied factors in the detection and recognition tasks. More details can be found in the “Methods” section. All the GLMM results can be found in the Supplementary Material 1. We establish the significance level at $p=0.05$. Neither the shape nor the location of the target had a significant influence on detection. The mean percentage of target **recognition** (*one of five possible responses; the participant could distinguish the shape of the stimulus*) for detected stimuli is 71.96% (± 12.36%). Recognition is calculated relatively to the detection percentage; a percentage of 100% recognition means that all detected visual targets have been correctly recognized. Different from detection, shape had a significant influence ($\beta =-0.311$, $t(293)=-3.324$, $p=0.001$) in recognition, with post-hoc Wilcoxon pairwise tests revealing that star shapes where better recognized. This may be related to the increased geometrical complexity of the star, which is the only non-convex shape in the stimuli.

### Experiment one: visual detection and recognition in the presence of temporally coherent auditory stimuli

In this experiment, each trial contained a single stimulus, which could be auditory-only, visual-only or bimodal (audiovisual). The background noise used in the baseline experiment was always present. A total of 54 stimuli were presented to each participant. Eighteen of them were visual-only, and followed the characteristics of the baseline experiment (we term them *visCond*). These stimuli also served as sentinels to make sure that all the participants had a good performance on detection and recognition tasks in the absence of confounding auditory stimuli. Another 18 stimuli were auditory-only, acting as distractors to make sure that participants would not expect a visual target to always appear in the presence of a sound. The last 18 stimuli were bimodal (*biCond*); these stimuli included both a visual target as in the baseline experiment, and a sound. Figure 2 illustrates the spatial and temporal layout of the experiment. No participant reported target detections in the auditory-only condition; in the following, we thus analyze the *visCond* and *biCond* conditions.

#### Influence of sound in detection and recognition

**Figure 3:**
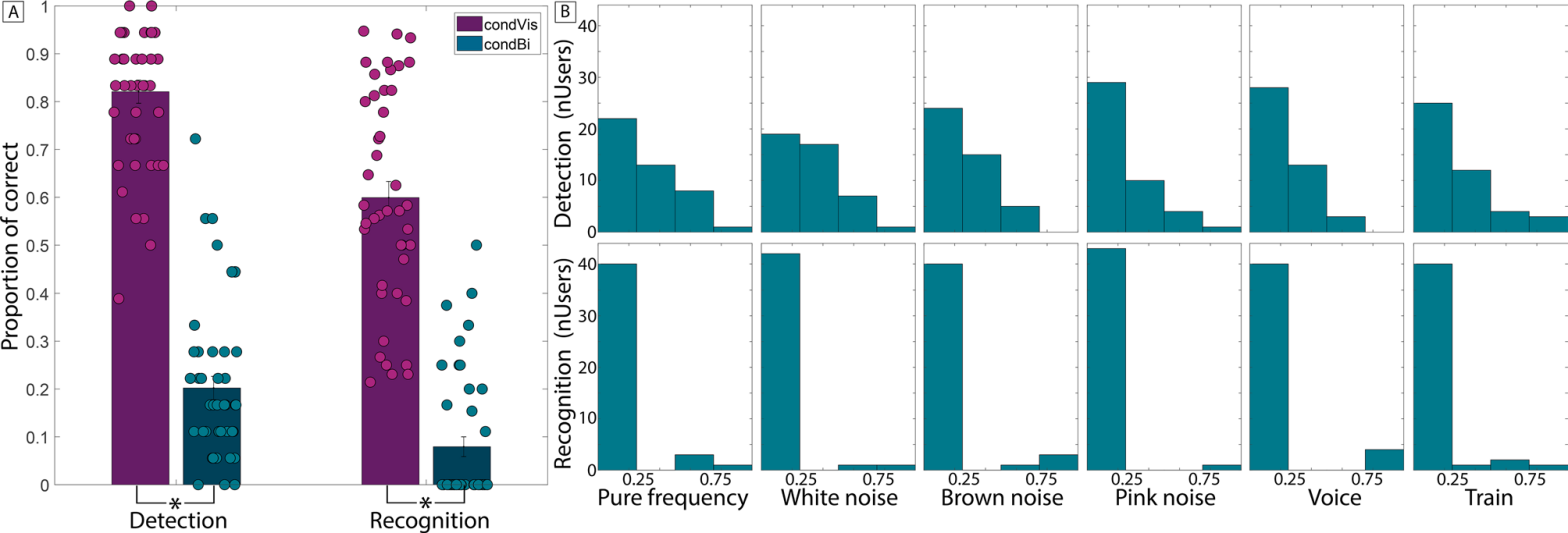
(**A**) Mean detection and recognition for *visCond* and *biCond* conditions. Error bars show 2 $\times$ SEM. Both detection and recognition are significantly lower for biCond stimuli (marked with an asterisk). Individual performance is shown as scattered points over the bars. (**B**) Mean detection (top row) and recognition (bottom row) histograms, by type of sound. Y-axes show the number of participants with a given performance rate (X-axes, from 0 to 1, divided in 4 bins). Note that a 0 detection rate for a given sound type implies a 0 recognition rate for the same sound type.

For the visual-only stimuli (*visCond*), the mean percentage of detection is 82.07% (± 4.81%), similar to the results from the baseline experiment. Adding sound (*biCond*) results in a large drop, yielding a mean percentage of detection of just 20.02% (± 4.86%). Similarly, recognition for *visCond* is 59.93% (± 6.76%), decreasing to only to 7.93% (± 4.12%) for *biCond*. This is shown in Fig. 3. A Wilcoxon signed rank test ($z=5.783$, $p<0.001$ for detection; $z=5.777$, $p<0.001$ for recognition) shows that both conditions are significantly different both in stimuli detection and recognition. In particular, we find a **decrease of both detection and recognition for** ***biCond*** **stimuli in relation to** ***visCond*** **stimuli**. A Wilcoxon rank sum test shows a significant difference ($z=7.919$, $p<0.001$) between *biCond* and the baseline experiment for both detection and recognition. Recognition drops from 71.96 to 59.93% for *visCond* compared to the baseline results. We hypothesize that this may be due to the greater cognitive load imposed on the participants, being exposed to three different stimuli conditions.

#### Effect of the different factors on detection and recognition

Here we analyze the influence of the different factors of the experiment (target location, target shape, sound location, and type of sound) on the detection and recognition tasks. As in the baseline experiment, the location of the visual target does not have a significant influence on detection nor recognition. The sound location does not have any significant influence either. Target shape has a significant influence ($\beta =-0.249$, $t(787)=-4.266$, $p<0.001$) only for *visCond* during recognition tasks, but not for *biCond*. The type of sound in the experiment has a significant influence ($\beta =0.658$, $t(613)=1.481$, $p=0.048$) on stimuli recognition (see Fig. 3). We found anecdotal evidence that pink and white noise had dominant effects in the degradation of visual performance, although these are not significant. A deeper study about which types of sound or which particular features (e.g., frequency content^21^) may have a deeper impact on visual performance degradation would be an interesting line for future work.

#### Analysis of gaze data

**Figure 4:**
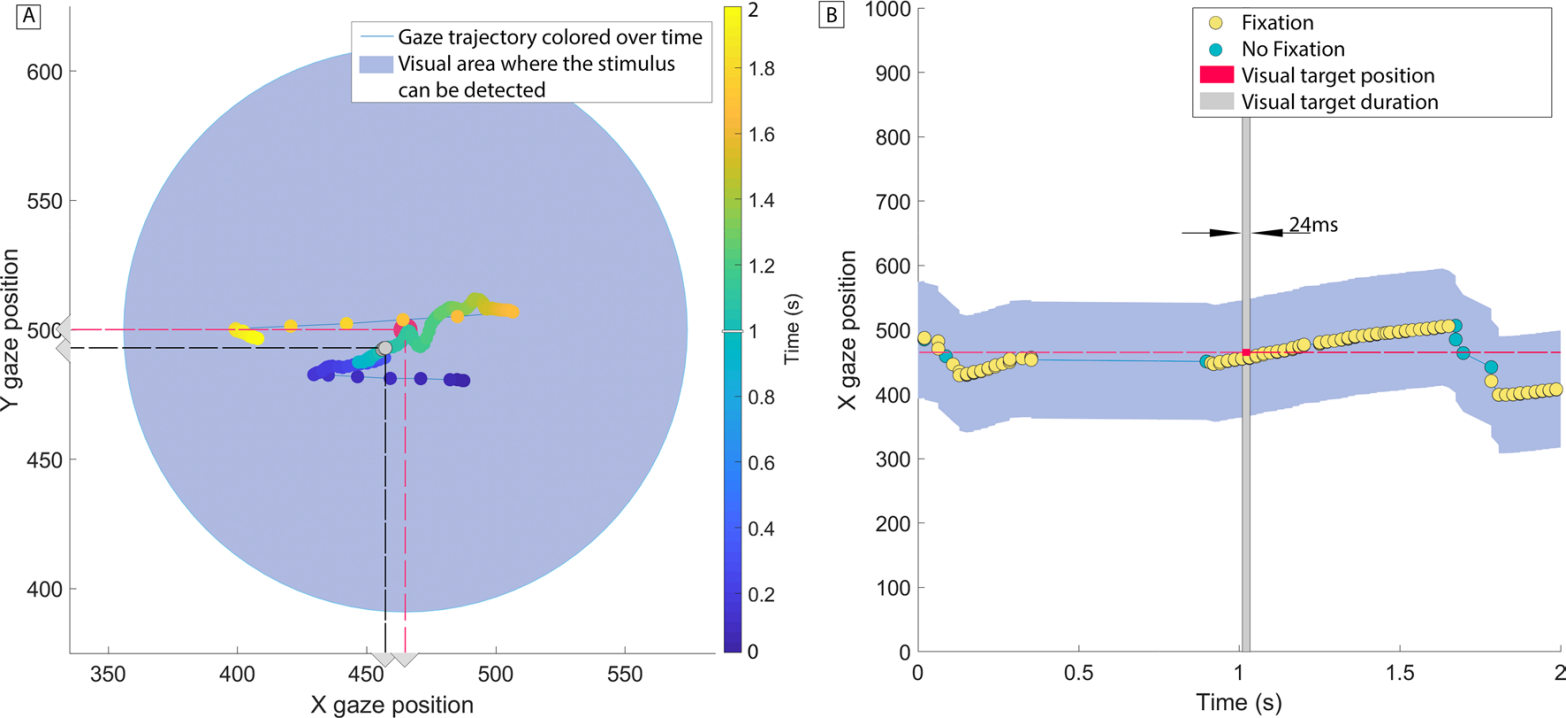
(**A**) Illustrative gaze behavior during presentation of a visual target that was not detected, corresponding to a *biCond* stimulus. The colored points represent gaze position during a 2-s window centered around the visual target onset. Target onset thus occurs at time $t=1$ s, and its spatial position is marked with a red point and associated red dashed lines. The blue-shaded area represents a region of 10$^{\circ }$ of visual angle^24^ centered around the target. Gaze position when the target is present is marked with a gray point and associated black dashed lines. Note that, despite gaze being very close to the target location during its onset, there is no detection. (**B**) 1D visualization of gaze position (only x coordinate) over time during the same trial presented in (**A**). Gaze samples corresponding to fixations are shown as yellow points, other gaze samples as blue points, the temporal interval during which the target is present is marked in gray, and the target position in x is marked by a dashed red horizontal line.

Auditory stimuli have the potential to trigger visual saccades^22^. Here we investigate saccades as a possible cause for the visual performance degradation effect. In particular, even though participants were explicitly told to ignore the sounds and focus on the visual targets, it is still possible that the auditory stimuli in the *biCond* condition were inducing a saccadic suppression effect, preventing the visual target from being seen. To analyze this, we leverage the data collected through the eye tracker and analyze gaze behavior around the visual target onset, focusing on the differences between *visCond* and *biCond* stimuli. However, accurate saccade detection is challenging, especially in our case where participants are allowed to move while wearing the VR headset. We thus study the differences in fixation rates between *visCond* and *biCond* stimuli as a more robust way of analyzing gaze behavior. We calculate fixation rates using fixation detection by two-means clustering^23^, which is robust in the presence of noise. We take into account a 2-s window centered around the visual target onset, and a region of interest of ten visual degrees^24^ around the position of the visual target (as shown in Fig. 4). We find that each participant fixates in that region 50.24% of the time on average in the *visCond* condition, and 49.13% in the *biCond* condition, with no significant difference between conditions ($z=0.671$, $p=0.502$, Wilcoxon signed rank test). If we reduce this window to the 400 ms around the visual target onset (the same 400 ms where sound is present in the *biCond* condition, as shown in Fig. 2), there is no significant difference either (72.40% vs 72.38% of the time on average, $z=0.933$, $p=0.3507$, Wilcoxon signed rank test). This suggests that the auditory part of *biCond* stimuli does not cause a significant change in gaze behavior. In particular, if saccadic suppression (a saccade triggered towards the sound source) was the underlying cause of the visual performance degradation, we would have expected to find a change in gaze behavior between *visCond* and *biCond*, with maybe a decrease of fixation time in the latter condition. In contrast, participants fixate similarly regardless of the presence of sound, while their visual performance varies significantly between *visCond* and *biCond*. This is confirmed by a qualitative analysis of gaze behavior, an example of which can be seen in Fig. 3. Visual performance degradation happens even when gaze is fixated close to the target location at its onset. Therefore, we believe that the degradation effect is not caused by oculomotor phenomena.

## Discussion

Interactions between the human visual and auditory systems are complex and not completely understood yet. Frens et al.^25^ showed that an auditory stimulus can improve performance of visual search tasks. At the same time, stimuli of one modality can alter^26,27^ or even suppress^7^ the perception of stimuli of another modality. Inspired by these works, we have investigated the auditory-triggered visual performance degradation effect under immersive and realistic viewing conditions, including natural exploratory behavior. We have verified that this crossmodal, sound-induced visual inhibitory effect exists in VR. In particular, we found that the effect is robust to different sound types, sound locations, as well as varying visual target shapes and locations along the FOV equator. The used VE also imposes a higher cognitive load on participants when compared to previous work. Even then, the degradation effect is robust to these potentially masking effects. Given that the visual degradation is robust to modifications of the four factors studied in this work, we hypothesize that the mechanism responsible for the degradation effect does not depend on the particular characteristics of the sound or the visual target to be inhibited, but rather encompasses a larger aspect of sensory perception.

We find a consistent and significant degradation of detection and recognition of visual targets in the presence of temporally congruent auditory stimuli. In their experiments about inhibitory audiovisual interactions, Hidaka and Ide^7^ used white noise bursts concurrent with the onset of their visual targets, under carefully controlled laboratory conditions including a chin rest and a conventional display. The authors reported a performance drop from 70.7% with visual-only stimuli to approximately 60% in the presence of sound. Our results show the same trend, and further suggest that, in the presence of higher cognitive loads, the degradation effect is more prominent, across a wide range of audiovisual stimuli. There is an important difference in the magnitude of the effect observed between our work and Hidaka and Ide’s. Possible reasons include the increased realism of our experiment, the increased complexity of the task (which includes binary detection as well as an additional five-level recognition task), and the fact that users were able to move during the experiment; all these may lead to higher cognitive loads. Besides cognitive load, the fact that sound is spatialized inside the virtual environment, and presented in rear space (in a spatially incongruent manner with respect to the visual targets) might further influence the effect magnitude. Additionally, Hidaka and Ide report a bigger effect when sounds were presented in an ipsilateral, spatially congruent manner. We did not find this effect with binaural spatialized sounds. Hence, their findings might be related with monoaural sounds rather than with the spatial congruency of visual and auditory stimuli.

We chose to use target stimuli that were not semantically related to the background scene, both in its visual and auditory aspects. We took a conservative approach, and designed the target visual stimuli as simple, white geometrical shapes that clearly stand out from the rest of the scene, to minimize the risk of fortuitous oversights. More contextually integrated visual stimuli may have lower detection percentages when compared to the visual targets used in this experiment.

Our analysis of gaze behavior shows that visual degradation occurs even in the presence of fixations and with gaze near or at the location of the visual target. Traditionally, sound has proven to increase performance of visual related tasks. For example, Corneil et al.^28^ show that saccades triggered by audiovisual stimuli have faster reaction times than those triggered by visual-only stimuli. However, other studies have also reported both facilitatory and inhibitory responses of audiovisual inputs, mostly depending on the spatiotemporal congruency of both modalities^15,29^. The more congruent the different modalities of the input stimuli presented are, the easier a facilitative integration will occur. On the other hand, if the stimuli are spatially or temporally incongruent, an inhibitory effect is more likely to occur. In our experiment, the visual and the auditory modalities of the stimuli (in *biCond*) were always presented in a temporally congruent and spatially incongruent manner. As to what is the underlying cause of the visual performance degradation effect, there are several possibilities, including oculomotor, neural and attentional effects. Our analysis of gaze behavior suggests that this phenomenon does not seem to be related to oculomotor effects. One possible explanation is that the auditory stimuli (a salient exogenous cue presented slightly before the visual target) is causing an involuntary shift of attention^30,31^. This attentional shift, either spatial^32^ or modal^33^, might in turn result in the degradation of visual performance or crossmodal deactivation of the visual input^34^. Note that in Hidaka and Ide’s work^7^ crossmodal attentional effects could not fully explain their findings, since the degradation effect was still present when the auditory stimuli were shown after the visual target. The authors concluded that the effect occurred based on neural interactions among auditory and visual modalities. One of the key differences in our experiment is that the auditory part of *biCond* stimuli is always shown 100 ms before the visual target onset, which may cause auditory stimuli to compete with the processing of visual stimuli^11^. Further studies are necessary in order to determine what is the exact cause behind the observed effect for both experimental conditions.

Besides increasing knowledge about the human visual system, leveraging visual performance degradation can also entail a direct benefit for several applications^35–38^. In particular, VR technology still faces challenging limitations that could be addressed with a deeper understanding of multimodal human perception. For instance, visual suppression has been used in conjunction with the *change blindness* phenomenon^39^ to introduce changes in the virtual world that go unnoticed by the users, allowing them to avoid obstacles in the physical world^40,41^. In general, a better understanding of the interplay of the different sensory modalities will lead to improved user experiences^42^. Apart from novel applications, we hope that our work can also motivate additional experiments to further study the scope of the visual performance degradation effect. We have shown how it affects both detection and recognition of a flashing visual target. Does it also affect the perceived motion of a dynamic visual target? Can we integrate inhibitory effects from different sensory modalities? It would also be interesting to analyze other sound properties: can we make the sound barely (if at all) noticeable while still degrading visual performance? Modeling and extending the parameter space of sounds that degrade visual perception might also give us some additional insights on the underlying perceptual mechanisms at work.

## Methods

### Participants and apparatus

Fifty-six participants took part in the experiments described in this work. Seven of them in the baseline experiment, and 49 in the main experiment (Exp. 1). The mean age was 24 years (± 3.21). Twenty of them were women. All of them had normal or corrected-to-normal vision and did not report hearing problems. Participants were not aware of the experiment’s goal. The visual and auditory stimuli were presented through an HTC Vive Pro VR headset with built-in headphones and a nominal field of view of 110$^{\circ }$ ($1440 \times 1600$ pixels resolution per eye and a framerate of 90 fps). A single computer was used, with an Intel i7-7700 processor at 3.6 GHz and 16 GB of RAM. The graphics card was an Nvidia 1060GTX (6GB of dedicated DDR5 memory). All the scenes were created using Unity 3D (2018 version) with the Vive VR plug-in on Windows 10. The VR headset included a Pupil-Labs eye tracker. This add-on eye tracker was used to record the participant’s gaze behavior through the experiment at 120 Hz, with an accuracy of 1$^{\circ }$ of visual angle.

Participants in Exp. 1 were presented with 18 audiovisual (*biCond*) stimuli, 18 visual-only (*visCond*) stimuli and 18 auditory-only stimuli. Participants in the baseline experiment (Exp. 0) were presented with 50 visual-only stimuli. Visual-only stimuli were the same for all participants in their respective experiments, while auditory-only stimuli were randomly chosen in Exp. 1. The auditory part of *biCond* stimuli was the same for all participants: Six different sounds in three possible locations each. The presentation of the different stimuli was randomized across participants to avoid order effects both in Exp. 0 and Exp. 1. We follow a conservative approach and consider for the analysis those participants with good detection and recognition percentages in *visCond* stimuli, setting a minimum detection and recognition threshold of 33% and 20%, respectively. As a result, only five participants were rejected from Exp. 1; their data was not considered for the analysis presented in the “Results” section.

### Visual stimuli (targets)

The visual targets consisted of five simple geometric white shapes with a gray outline, as shown in Fig. 1. In order of increasing complexity: circle, square, rhombus, pentagon and star. They were chosen not to have any semantic meaning compared to the visual background scene. The target size is 1$^{\circ }$ of visual angle. Visual targets remain for 24 ms in the participant’s FOV. Both the target size and its duration had been fixed following Hidaka and Ide’s work^7^. In our experiment, the target could appear randomly at one of three different locations, always at the same latitude (FOV equator line): FOV center, 4$^{\circ }$ of visual angle to the left or 4$^{\circ }$ of visual angle to the right of it. These stimuli were used both in Exp. 1 and in the baseline experiment, where their visibility was assessed. Visual-only (*visCond*) stimuli were maintained in Exp. 1 as sentinels.

### Auditory stimuli

Auditory stimuli included six different sounds inspired by previous literature. *Pure frequency:* We are not used to pure frequency sounds in nature^2^. Being less common, this sound could deviate the participant’s attention from the visual stimuli. *White noise:* This is the sound used by Hidaka and Ide^7^. It has proven to degrade performance in visual recognition tasks in traditional displays. *Brown noise:* Random changes between tones can stand out from uniform noises^2^. *Pink noise:* Pink noise is known to trigger an acoustic reflex response that protects the eardrum from loud noises^43^. Given the relationship between visual and auditory neural processing, we hypothesized that pink noise could also have an inhibitory effect on visual stimuli. *Survival sound:* Critical sounds for our survival also stand out, especially if they come from outside our FOV^2^. In particular we used a train horn in Exp. 1. *Human voice:* It has been shown that human voices draw our attention powerfully^2^. The duration of each sound was 400 ms, to allow for the more complex sounds to play completely. Sounds were spatially located at random in one of three possible locations, always at 0.2 m (Unity distance) from the head: directly behind the participant’s head, shifted to the right (50$^{\circ }$ rotation from the center of the head’s position) or to the left (also 50$^{\circ }$), always outside their FOV. Auditory-only stimuli served as distractors, to avoid an association of the visual target appearance with the auditory stimuli onset.

### Audiovisual stimuli

Audiovisual stimuli were created by presenting simultaneously an auditory stimulus and a target. As shown in Fig. 2B, the auditory stimuli start playing 100 ms before the visual stimuli onset. Every participant was presented with the six possible sounds in the three described locations, making a total of 18 different audiovisual stimuli. The visual part of each stimuli was chosen pseudo-randomly (as close to a uniform distribution as possible) across participants. Figure 2A shows all the possible locations of the bimodal stimuli.

### Procedure of experiment one

(The baseline experiment procedure is the same, but with visual stimuli only.) The participants were located inside a virtual scene that resembled a living room, shown in Fig. 1. They could freely move in a physical space of 4 $\times$ 1.5 m with a 1:1 mapping between the real and virtual spaces. Before starting the experiment, the participants were shown the same room without furniture so that they got used to the VR headset and the VE. Participants were informed of their task until they declared they had understood it. Simple geometric shapes would appear and disappear in front of them randomly throughout the experiment; each time they detected one such shape, they had to notify the experimenter. The experimenter would then show them a question within the VE: *What did you see?* When the participant answered, the experimenter would log the answer and the experiment resumed. No new stimuli appeared until the participant had answered the question. This was an open-ended task, as the participants did not know *a priori* what specific shapes could appear during the experiment. If the participant **detected** the onset of a visual stimulus but did not **recognize** its specific shape they still had to notify it. The participants were also told that they would hear several sounds throughout the experiment, but that they had to stay focused on the appearance of the visual target. There was an additional background sound played throughout the whole experiment: the sound of a park through an open window and a news podcast that played through one of the speakers near the TV. The intention of this background sound was to increase the scene complexity and realism, as well as to avoid the auditory stimuli being the only sounds in the scene.

Throughout the experiment, the three different types of stimuli (visual, auditory and audiovisual) appeared in random order with a random in-between interval that varied from 5 to 10 s. The experiment took 15–20 min, including the initial explanation and the questionnaires that the participants filled before and after the experiment. The participants were informed to stop the experimenter if they felt any kind of sickness or discomfort during the experiment (none did). Before they started to use the VR headset, participants filled a questionnaire with sociodemographic questions including age, gender, and previous experience with VR. None of the sociodemographic factors had an influence on the obtained results. After the experiment had concluded, there was a short debriefing in which they filled a set of questions about the experiment (*Did you see or hear something remarkable?*, *Did you feel any discomfort?*, *Do you want to say something else about the experiment?*). None of the participants experienced sickness or discomfort after the experiment. Six of them reported either the train horn or the human voice were surprising at least the first time they appeared in the experiment. Nine found the task *interesting* or *engaging*.

### Statistical analysis

A GLM assumes that the measured data samples are independent. In our case, we cannot assume that the samples are independent, since each participant was measured several times under different conditions. Using a GLMM we can account for mixed effects, and therefore account at the same time for both the fixed effects of our variables and the random effect corresponding to user variability. The dependent variable was binary (for detection) or categorical (for recognition). The independent variables in both cases were the visual target shape, the visual target location, the sound type, and the sound location; they were set as fixed effects. Different participants (in particular, the recorded subject ID) were considered as random effects. We used *Matlab* fitglme function with a logit link function.

### Ethics statement

The experiments were conducted in accordance with the guidelines and regulations of Universidad de Zaragoza (Spain). Our experimental protocols comply with the requirements approved by the Consejo de Gobierno (Government Council) of Universidad de Zaragoza. Written informed consent was obtained from participants before experiments began, and particular attention was paid to ensure that research data could be curated in an anonymized manner. At the outset of the experiment it was made clear to participants that they participated voluntarily and that they had the right to withdraw from the research at any time without giving a reason.

## Supplementary information

Supplementary file 1
