# Supplementary material for: Auditory stimuli degrade visual performance in virtual reality
Source: Sci Rep. 2020 Jul 23;10:12363. doi: 10.1038/s41598-020-69135-3 (PMC7378072; doi:10.1038/s41598-020-69135-3)
Supplement: Supplementary file 1 [file 41598_2020_69135_MOESM1_ESM.pdf]

Supplementary material:

Auditory Stimuli Degrade Visual Performance in Virtual Reality

Sandra Malpica, Ana Serrano, Diego Gutierrez and Belen Masia

Universidad de Zaragoza, I3A

This document offers additional information and details on the following topic:

- Additional details on the statistical analysis (Results Section in the paper).

A Additional details on the statistical analysis

In the following section we provide additional information about the generalized linear mixed models (GLMM) used to analyze the statistical influence of the studied factors (target shape, sound type, sound location, target location as fixed-effects; subject ID as a random-effect) in the collected data from our experiments (two response variables, detection and recognition, binary and categorical respectively). We used Matlab `fitglme` function with the `logit` link function.

A.1 Baseline experiment: detection


```
Fixed effects coefficients (95% CIs):
    Name                          Estimate     SE         tStat       DF     pValue        Lower       Upper
    '(Intercept)'                   1.9717       0.327      6.0295    334    4.3555e-09      1.3284     2.6149
    'target shape'               -0.058613     0.11778    -0.49763    334       0.61907     -0.2903    0.17308
    'target position'              0.12855     0.20748     0.61959    334       0.53595    -0.27957    0.53667

Random effects covariance parameters:
Group: userID (7 Levels)
    Name1                 Name2                 Type         Estimate
    '(Intercept)'         '(Intercept)'         'std'        8.5405e-05

Group: Error
    Name                        Estimate
    'sqrt(Dispersion)'          1
```

A.2 Baseline experiment: recognition

Fixed effects coefficients (95% CIs):

Name	Estimate	SE	tStat	DF	pValue	Lower	Upper
'(Intercept)'	1.4481	0.27554	5.2556	293	2.8465e-07	0.90583	1.9904
'target shape'	-0.31148	0.093721	-3.3235	293	0.0010019	-0.49593	-0.12703
'target location'	0.070501	0.16242	0.43407	293	0.66456	-0.24915	0.39015

Random effects covariance parameters:
Group: userID (7 Levels)

Name1	Name2	Type	Estimate
'(Intercept)'	'(Intercept)'	'std'	5.3984e-13

Group: Error

Name	Estimate
'sqrt(Dispersion)'	1

A.3 Experiment one: *visCond* detection

Fixed effects coefficients (95% CIs):

Name	Estimate	SE	tStat	DF	pValue	Lower	Upper
'(Intercept)'	1.4908	0.22253	6.6992	787	3.9913e-11	1.054	1.9276
'target shape'	-0.020626	0.06605	-0.31228	787	0.75491	-0.15028	0.10903
'target location'	0.021187	0.11443	0.18516	787	0.85315	-0.20343	0.2458

Random effects covariance parameters:
Group: userID (44 Levels)

Name1	Name2	Type	Estimate
'(Intercept)'	'(Intercept)'	'std'	0.77266

Group: Error

Name	Estimate
'sqrt(Dispersion)'	1

A.4 Experiment one: *visCond* recognition

visCond recognition

Fixed effects coefficients (95% CIs):

Name	Estimate	SE	tStat	DF	pValue	Lower	Upper
'(Intercept)'	0.29588	0.23095	1.2811	787	0.20052	-0.15747	0.74923
'target shape'	-0.2487	0.058297	-4.2662	787	2.2307e-05	-0.36314	-0.13427
'target location'	-0.068519	0.10075	-0.68011	787	0.49663	-0.26628	0.12925

Random effects covariance parameters:
Group: userID (44 Levels)

Name1	Name2	Type	Estimate
'(Intercept)'	'(Intercept)'	'std'	1.0773

Group: Error

Name	Estimate
'sqrt(Dispersion)'	1

A.5 Experiment one: *biCond* detection

Fixed effects coefficients (95% CIs):

Name	Estimate	SE	tStat	DF	pValue	Lower	Upper
'(Intercept)'	-1.4579	0.27886	-5.228	787	2.1003e-07	-2.0052	-0.91066
'target shape'	0.0011929	0.060857	0.019601	787	0.98437	-0.11823	0.12062
'sound type'	0.070458	0.051477	-1.3687	787	0.1714	-0.17148	0.030561
'sound location'	0.12425	0.1084	1.1462	787	0.25201	-0.088484	0.33698
'target location'	-0.12446	0.10836	-1.1485	787	0.25103	-0.33711	0.088195

Random effects covariance parameters:

Group: userID (44 Levels)

Name1	Name2	Type	Estimate
'(Intercept)'	'(Intercept)'	'std'	0.09101

Group: Error

Name	Estimate
'sqrt(Dispersion)'	1

A.6 Experiment one: *biCond* recognition

Fixed effects coefficients (95% CIs):

Name	Estimate	SE	tStat	DF	pValue	Lower	Upper
'(Intercept)'	-4.5449	0.86972	-5.2257	613	2.3814e-07	-6.2529	-2.8369
'target shape'	-0.31147	0.26125	-1.1922	613	0.23364	-0.82453	0.20159
'sound type'	0.65811	0.4836	1.481	613	0.04813	0.08524	1.3215
'sound location'	0.16714	0.17433	0.95874	613	0.33807	-0.17522	0.5095
'target location'	-0.081096	0.35234	-0.23017	613	0.81804	-0.77303	0.61084

Random effects covariance parameters:

Group: userID (44 Levels)

Name1	Name2	Type	Estimate
'(Intercept)'	'(Intercept)'	'std'	0.00012517

Group: Error

Name	Estimate
'sqrt(Dispersion)'	1
